# Experiences of trauma and psychometric properties of the Life Events Checklist among adults in Uganda

**DOI:** 10.1371/journal.pone.0298385

**Published:** 2024-04-30

**Authors:** Zahra Morawej, Supriya Misra, Amantia A. Ametaj, Anne Stevenson, Joseph Kyebuzibwa, Bizu Gelaye, Dickens Akena

**Affiliations:** 1 Department of Psychiatry, Faculty of Medicine, Hubert Kairuki Memorial University, Dar es Salaam, Tanzania; 2 Department of Psychiatry, School of Medicine, College of Health Sciences, Makerere University, Kampala, Uganda; 3 Department of Public Health, San Francisco State University, San Francisco, CA, United States of America; 4 Department of Epidemiology, Harvard T. H. Chan School of Public Health, Boston, MA, United States of America; 5 Stanley Center for Psychiatric Research at Broad Institute of MIT and Harvard, Cambridge, MA, United States of America; 6 Department of Psychiatry, Faculty of Medicine and Health Sciences, Stellenbosch University, Cape Town, South Africa; 7 Department of Psychiatry, Massachusetts General Hospital, Boston, MA, United States of America; 8 Department of Psychiatry, Harvard Medical School, Boston, MA, United States of America; University of Toronto, CANADA

## Abstract

Exposure to potentially traumatic events (PTE) is common and increases an individual’s risk of developing post-traumatic stress disorder (PTSD) and other psychiatric disorders. PTEs can be screened with the Life Events Checklist for DSM 5 (LEC-5). However, the psychometric properties of the LEC-5 have never been assessed in Uganda. We aimed to estimate the prevalence of PTEs and evaluate the factor structure of the LEC-5 in a sample of N = 4,479 Ugandan adults between February 2018 –March 2020. We used the phenotyping data from a case-control study (NeuroGAP-Psychosis) in Uganda investigating the genetic and environmental risk factors for psychosis spectrum disorders with 4,479 participants (2,375 cases and 2,104 controls). Prevalence for PTEs was determined for all participants and by case-control status. The factor structure of the LEC-5 was assessed using an exploratory factor analysis (EFA) and a confirmatory factor analysis (CFA). The overall prevalence of exposure to one or more types of PTEs was 60.5%. Cases reported more frequency of exposure to PTEs than controls (64.2% vs 55.4%; p<0.001). The most frequently endorsed traumatic event was physical assault (22.8%), while exposure to toxic substances was the least endorsed (1.7%). There were several differences among the types of events experienced between cases and controls, including cases reporting more experiences of physical (28.6% vs. 16.2%, p<0.001) and sexual assault (11.5% vs. 5.0%, p<0.001) than controls. The EFA yielded a six-factor model that explained 49.8% of the total variance. The CFA showed that a theoretical seven-factor model based on the South African Stress and Health survey was a better fitting model (CFI = 0.935; TLI = 0.908; RMSEA = 0.026) than the EFA. This study revealed a high prevalence of PTEs among cases and controls, and the LEC-5 was found to have good psychometric properties among Ugandan adults.

## Introduction

Traumatic events such as serious accidents, physical and sexual abuse, and sudden deaths of loved ones are common occurrences globally [1–3]. The World Mental Health Surveys recently assessed the prevalence of 29 types of traumatic experiences across 24 countries and found that 70.4% of respondents experienced at least one traumatic event in their lifetime [2, 3]. In high-income countries like Norway, at least one serious lifetime event was reported by 85% of men and 86% of women [4]. Studies focusing on trauma exposure in sub-Saharan Africa have yielded variable findings. For example, a study among urban schools in Kenya and South Africa found that more than 80% of participants reported either directly experiencing or witnessing at least one traumatic event [5]. Another study in Kenya reported that 64.4% of participants had experienced at least one potentially traumatic event [6]. A study in Ethiopia noted that the prevalence of experiencing a potentially traumatic event was 48.7% [7]. In Uganda, most of the studies on traumatic events have focused on conflict areas such as northern Uganda. For example, the Wayo-Nero study in northern Uganda found that 80.8% of participants experienced at least one war-related traumatic event (e.g., war-related physical trauma, war-related psychological trauma, and war-related sexual trauma), and 47.2% experienced at least three war-related traumatic events [8]. However, there is limited information on how measures perform for assessing traumas in Uganda.

Potentially traumatic events (PTEs) are associated with many serious adverse mental health effects. It is estimated that about 15–25% of those who experience a traumatic event will go on to develop post-traumatic stress disorder [3] and other psychiatric conditions, including depressive disorders, substance use disorders, anxiety disorders, and psychotic disorders, among others [9, 10]. Studies have also shown that people with psychiatric disorders are more likely to have experienced PTEs [11, 12]. Psychiatric conditions impair functioning and add to the global burden of disease [13]. Therefore, screening for PTEs is important to prevent the development of psychiatric disorders and provide timely treatment.

Given the high prevalence and impact of traumatic events, it is imperative to have trauma assessment tools that can be utilized in clinical settings as well as within the general population. Individuals with psychotic disorders, in particular, are at an increased risk of experiencing traumatic events, and up to 25–50% of patients with psychotic spectrum disorders in high-income countries have co-occurring PTSD [14–16]. Additionally, high rates of traumatic events and PTSD, particularly interpersonal traumas, have been reported for individuals with psychotic disorders in some low- and middle-income countries, including India [17, 18]. Therefore, understanding traumatic events relevant to patients with psychotic disorders may be especially important for screening and treating these patients with the proper tools. Certain PTEs may group together and have a higher chance of leading to certain symptoms (e.g., interpersonal PTEs and psychotic disorders). The relationship between these clusters may be helpful to explore their relationship to psychopathology [19, 20]. Measuring and modeling trauma exposure as composite variables may be helpful to this end by aggregating two or more highly related traumatic events [21].

Multiple assessment tools are available for use in evaluating PTEs. The Life Events Checklist (LEC-5; Weathers et al., 2013) for the *Diagnostic and Statistical Manual of Mental Disorders*, *5*^*th*^ *edition* (DSM-5; [22] is a widely used measure. The LEC has previously been applied in many cultural settings, including Africa [23, 24]. However, to our knowledge, it has not been used in the Ugandan setting before. While the LEC is one of the most widely used instruments to assess exposure to PTEs [25], only a handful of studies have evaluated the factor structure of the scale [6, 7, 26, 27] including our colleagues’ findings from the larger parent study for Ethiopia, Kenya, and South Africa [6, 7, 27]. These studies showed good factorial validity for the LEC-5 [6, 7, 26, 27]. The factor structure of most tools for assessing traumatic events, including LEC-5, have not been assessed for use in the Ugandan setting.

This study aimed to determine the prevalence of PTEs for Ugandan adults, including specifically for cases (i.e., individuals with a psychosis-related diagnosis) and controls (i.e., individuals in a general medical setting) and to evaluate the factor structure of the LEC-5 in clinical settings in Uganda with data from a large neuropsychiatric study. Understanding the prevalence of PTEs between cases and controls is essential for clinical relevance and can guide interventions, treatments, and strategies aimed at addressing the specific needs of each group.

## Materials and methods

### Study setting and participants

This study was conducted as part of the Neuropsychiatric Genetics of African Populations-Psychosis (NeuroGAP-Psychosis) program in Ethiopia, Kenya, South Africa, and Uganda. These analyses focus on the Ugandan sample. NeuroGAP-Psychosis is a case-control study aimed at identifying genetic and environmental risk factors contributing to the risk of psychotic disorders in African populations [28].

Study participants were recruited as outpatients from psychiatric and general medical hospitals across Uganda. The inclusion criteria for cases required a clinical diagnosis of a psychosis spectrum disorder: schizophrenia, schizoaffective disorder, bipolar disorder, mania “not otherwise specified,” or psychosis “not otherwise specified.” Cases were patients receiving outpatient care in the form of prescription refill and follow-up or those discharged from in-patient care and awaiting relatives to take them home. Controls were individuals without any history of psychosis spectrum disorders from a general medical setting. General inclusion criteria consisted of adults ages 18 or over who were fluent in one of the languages in which the study was conducted: Acholi-Luo, English, Luganda, Lugbara, or Runyankole. Exclusion criteria included acute levels of alcohol or substance abuse (e.g., under inpatient psychiatric or medical care for one of these conditions). We used an adapted version of the University of California, San Diego Brief Assessment of Capacity to Consent (UBACC; [29] to ensure that all participants (cases and controls) had sufficient capacity and autonomy to participate in the study. All participants provided informed consent to participate in the study.

### Procedure

Participants from Uganda were enrolled in this study from February 2018 to March 2020. They were recruited from five health centers across the four regions of Kampala, Arua, Mbarara, and Gulu: Butabika National Mental Health Referral Hospital and Naguru Hospital (controls only) in Kampala region, Arua Regional Referral Hospital, Mbarara Regional Referral Hospitals, and Gulu Regional Referral Hospitals. Data across sites was collected at one point in time. Research staff received training on data collection, ethical considerations in research, consenting process, interview techniques, questionnaire administration, and use of the data collection tablets. Trained research assistants administered the LEC-5 upon consent. We obtained ethical approval from all participating sites: Makerere University School of Medicine Research and Ethics Committee (SOMREC #REC REF 2016–057) and Uganda National Council for Science and Technology (UNCST #HS14ES) in Uganda, and the Harvard T.H. Chan School of Public Health (#IRB17-0822) in the United States. More detailed information about NeuroGAP-Psychosis can be found in the study protocol [28].

### Measures

#### Life Events Checklist for DSM-5 (LEC-5)

The LEC-5 is a 17-item self-report scale used to assess the lifetime prevalence of exposure to PTEs [30]. It includes PTEs that are experienced directly (“happened to me”) or witnessed. The original version of DSM-IV was validated among college students and combat veterans in the United States [31]. Minimal revisions were made to the LEC-5 when the DSM-5 was released, and the scale was not expected to have notable psychometric differences compared to the prior version [30]. We limited our analysis to exposure to 16 types of PTEs. We excluded the last item on the scale, “any other stressful experience” since it is a catchall category that would not conceptually adhere to a shared factor with other items.

#### Sociodemographic characteristics

All participants enrolled in the study also provided information on several demographic variables, including sex at birth, age, the highest level of education, marital status, and current living arrangement.

### Data analytic plan

Stata 15 was used for all data analyses [32]. First, frequency distributions of sociodemographic characteristics, individual PTEs, and experiencing any PTEs were calculated. Student’s t-test for continuous variables and Chi-square test for categorical variables were used to test bivariate differences between cases and controls. We initially examined the factor structure of the LEC-5 using exploratory factor analysis (EFA) on a randomly split-half sample from our data. Eigenvalues and a scree plot served to identify the number of factors to retain for the EFA. Factors with eigenvalues >1 were retained, followed by varimax rotation. Items with rotated factor loadings ≥ 0.3 were considered as loading on a factor with the highest values used to assign each item to a single factor for cross-loading items. Items < 0.3 were not regarded as related to a factor and were dropped.

Subsequently, a confirmatory factor analysis (CFA) was conducted to compare the findings obtained from our EFA and two alternative models based on existing literature. The first model, corresponding to the outcomes of our EFA, was evaluated using the second half of the split sample to test the EFA-derived results. The two additional models were assessed using the full dataset. These included: (a) a six-factor model, derived from the EFA of the Life Events Checklist (LEC) conducted in South Korea [26], which concurred with the factor structure derived from a 27-item module of the World Health Organization Composite International Diagnostic Interview (CIDI) incorporated in the World Mental Health (WMH) Survey [2], and (b) a theoretical seven-factor model of the same 27-item module from the WHO CIDI, as utilized in the South African Stress and Health Survey [33, 34]. It is noteworthy that, although the WHO module encompassed a larger item set, the LEC-5 items could be categorized into six overarching domains, namely war events, physical violence, sexual violence, accidents, network events, and witnessing death. Notably, the LEC-5 excluded the item pertaining to the “unexpected death of loved one” but did incorporate a separate item denoted as “severe suffering.” Measurement error was assumed to be independent, however, latent variables for all the three models were allowed to be correlated with one another based on the expected relationship between traumatic events. The first item for each latent factor served as the marker item, and the reliability for single item indicators was set at 0.80.

To compare the three CFA models, the following standard indices of model fit were employed: (1) standardized root mean square residual (SRMR) close to 0.08 or below; (2) root mean square error of approximation (RMSEA) close to 0.06 or below; (3) comparative fit index (CFI) close to 0.90 or above; (4) Tucker-Lewis index (TLI) close to 0.90 or above [35].

## Results

This study involved a total of 4,479 participants (2,375 cases and 2,104 controls), with a mean age of 35.9 years (SD = 12.2), the majority (76.6%) of whom were aged below 45 years. There were slightly more female (54.1%) than male participants, and the majority of the sample (74.5%) had either some primary or some secondary education (Table 1).

**Table 1:** Ugandan sample demographics (N = 4479) *.

	Count	%
Cases	2375	53.0
Age (*Mean*, *SD*)	*35*.*9*	*12*.*1*
Sex (*%*)		
Female	2424	54.1
Male	2055	45.9
Age categories (*%*)		
18–29	1544	34.5
30–44	1886	42.1
45–64	953	21.3
65+	96	2.1
Marital status (*%*)		
Single	1593	35.6
Married or cohabitating	1906	42.6
Widowed	245	5.5
Divorced or separated	732	16.3
Level of education (*%*)		
No formal education	186	4.2
Primary	1427	31.9
Secondary	1908	42.6
University	955	21.3
Living arrangements (*%*)		
Alone	603	13.5
With parents	1125	25.1
With spouse or partner	1648	36.8
With friends or other relatives	1095	24.4
Unknown	8	0.2

* *Note*: Counts may not add up to the total due to missing information for some participants

Overall, 60.5% of participants reported experiencing one or more PTEs, while 19.3% endorsed three or more PTEs (Table 2). The most frequently endorsed PTEs were physical assault (22.8%) and exposure to combat/war-zone (15.1%). Exposure to toxic substances was the least endorsed item (1.7%). The frequency of traumatic events stratified by case-control status is also presented in Table 2. Cases (64.2%) were more likely to have experienced one or more PTEs compared to controls (55.4%, p < 0.001). Cases were also more likely than controls to report experiencing serious accidents (8.9% vs. 6.6%; p = 0.005), physical assault (28.6% vs 16.2%; p < 0.001), weapon assault (12.8% vs. 10.1%; p = 0.006), sexual assault (11.5% vs. 5.0%; p < 0.001), and unwanted sexual experiences (7.3% vs. 5.1%; p = 0.004). Controls were more likely than cases to have experienced illness/serious injury (11.4% vs. 8.1%; p < 0.001) and sudden violent or accidental death of a close loved one (12.9% vs. 11.3%; p = 0.039).

**Table 2:** Frequency of traumatic events according to case control status.

	Total (N = 4479)	Cases (N = 2375)	Controls (N = 2104)	
	n (%)	n (%)	n (%)	p-value
Frequency of events				
Any event	2710 (60.5)	1525 (64.2)	566 (55.4)	<0.001*
None	1769 (39.5)	850 (35.8)	456 (44.8)	<0.001*
One	1108 (24.8)	595 (25.1)	250 (24.6)	
Two	731 (16.3)	418 (17.6)	139 (13.7)	
Three	408 (9.1)	252(10.6)	87 (8.6)	
Four or more	458 (10.2)	259 (10.9)	86 (8.5)	
Type of event				
Natural disaster	136 (3.0)	70 (3.0)	66 (3.1)	0.706
Fire/explosion	345 (7.7)	183 (7.7)	162 (7.7)	0.994
Transport accident	434 (9.7)	244 (10.3)	190 (9.1)	0.165
Serious accident	348 (7.8)	210 (8.9)	138 (6.6)	0.005*
Toxic substance	74 (1.7)	38 (1.6)	36 (1.7)	0.766
Physical assault	1019 (22.8)	679 (28.6)	340 (16.2)	<0.001*
Weapon assault	516 (11.5)	202 (12.8)	213 (10.1)	0.006*
Sexual assault	378 (8.5)	274 (11.5)	104 (5.0)	<0.001*
Unwanted sexual experience	280 (6.3)	172 (7.3)	108 (5.1)	0.004*
Combat/war-zone	675 (15.1)	358 (15.1)	317 (15.1)	0.989
Captivity	136 (3.0)	64 (2.7)	72 (3.4)	0.154
Illness/injury	431 (9.6)	191 (8.1)	240 (11.4)	<0.001*
Severe suffering	252 (5.6)	131 (5.5)	121 (5.8)	0.724
Sudden violent death	538 (12.0)	267 (11.3)	271 (12.9)	0.089
Sudden accidental death	394 (8.8)	189 (8.0)	204 (9.7)	0.039*
Caused serious				
harm/death	135 (3.0)	90 (3.8)	45 (2.1)	0.001*

*Note*: Chi-square test p-value is denoted for number of events. Trend test p-value is noted for differences between cases and controls for “any event” and for each different type of event.

*denotes significance of at least p < 0.05

The EFA analyses conducted on a random split-half of our sample resulted in a five-factor solution with item loadings varying from 0.30 to 0.82 (Table 3). The five factors were labeled as follows based on item grouping: sexual violence, war and death, physical violence, environment and war-related trauma, and accidents and injury. Three items (combat, severe suffering, and fire/explosion) cross-loaded on more than one factor but were assigned to the factor with the higher loading. Lastly, the item serious accident had a factor loading below the 0.3 cut-off and was not assigned to any of the five factors from the EFA.

**Table 3:** Principal component analysis with varimax rotation of Life Events Checklist for DSM-5 (LEC-5).

LEC Item	Factor Loading				
	1	2	3	4	5
**Factor 1: Sexual Violence**					
8. Sexual Assault	0.82				
9. Other Unwanted	0.81				
Sexual Experience					
**Factor 2: War and Death**					
10. Combat/War Zone		0.41		0.39	
14. Violent Death		0.72			
15. Accidental Death		0.67			
**Factor 3: Physical Violence**					
6. Physical Assault			0.61		
7. Weapon Assault			0.58		
13. Severe Suffering		0.30	0.36		
16. Caused Harm/Death			0.60		
**Factor 4: Environment and War Related**					
1. Natural Disaster				0.52	
5. Toxic Substance				0.62	
11. Captivity				0.55	
**Factor 5: Accident and Injury**					
2. Fire/Explosion				0.35	0.41
3. Transport Accident					0.64
12. Life-Threatening					0.66
Illness/Injury					
*4. Serious Accident	-	-	-	-	-
**Eigenvalue**	1.62	1.44	1.42	1.400	1.2020
**% Variance**	10.07	8.99	8.88	8.77	7.50
**Total: 44.21**					

Note: Loadings smaller than 0.3 are not displayed. * Item 4 (“serious accidents”) had a loading below 0.3 and was not assigned to any of the factors.

**Fig 1:**
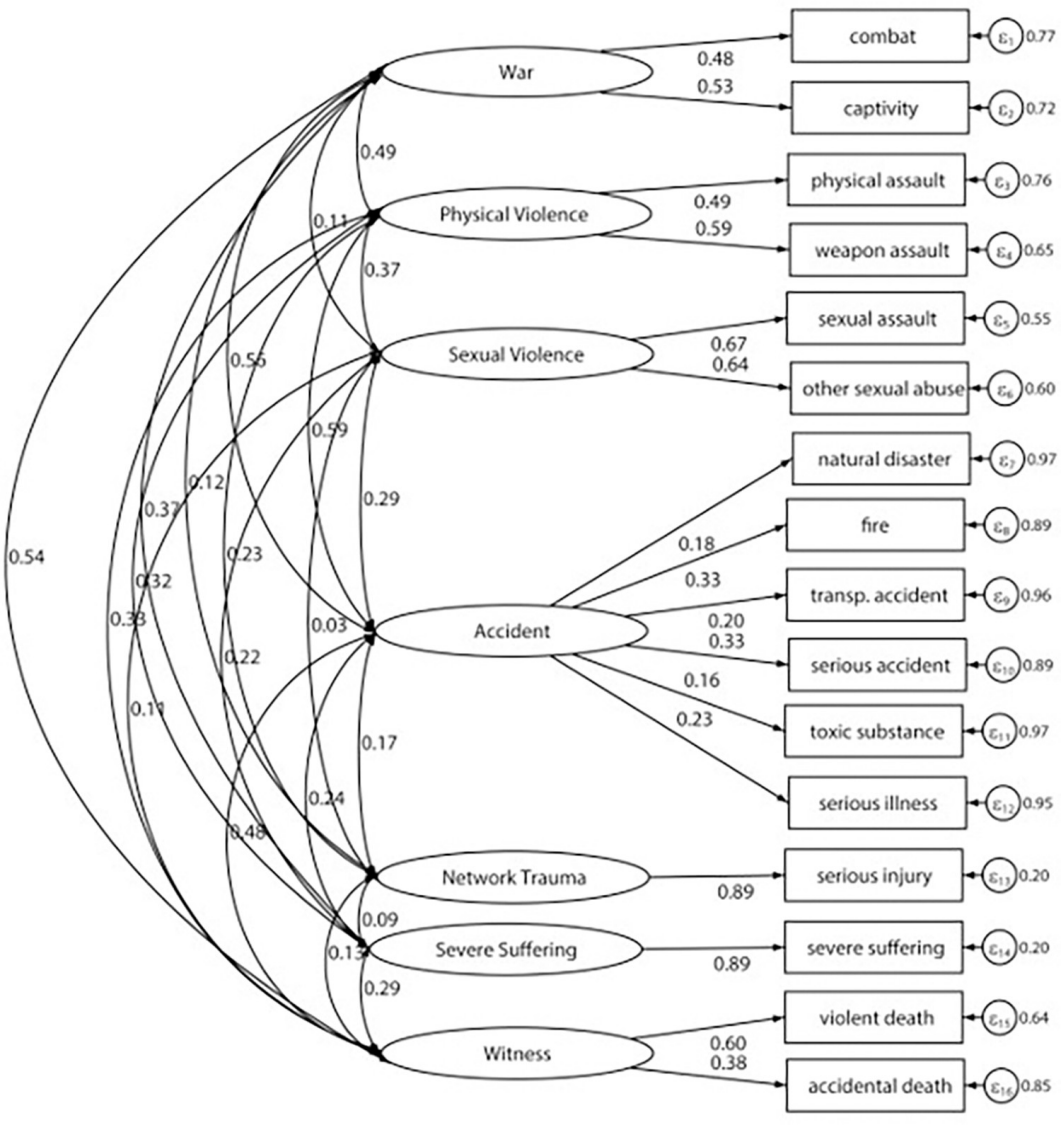
Final seven-factor model selected for Life Events Checklist for DSM-5 (LEC-%) in Uganda.

**Fig 2:**
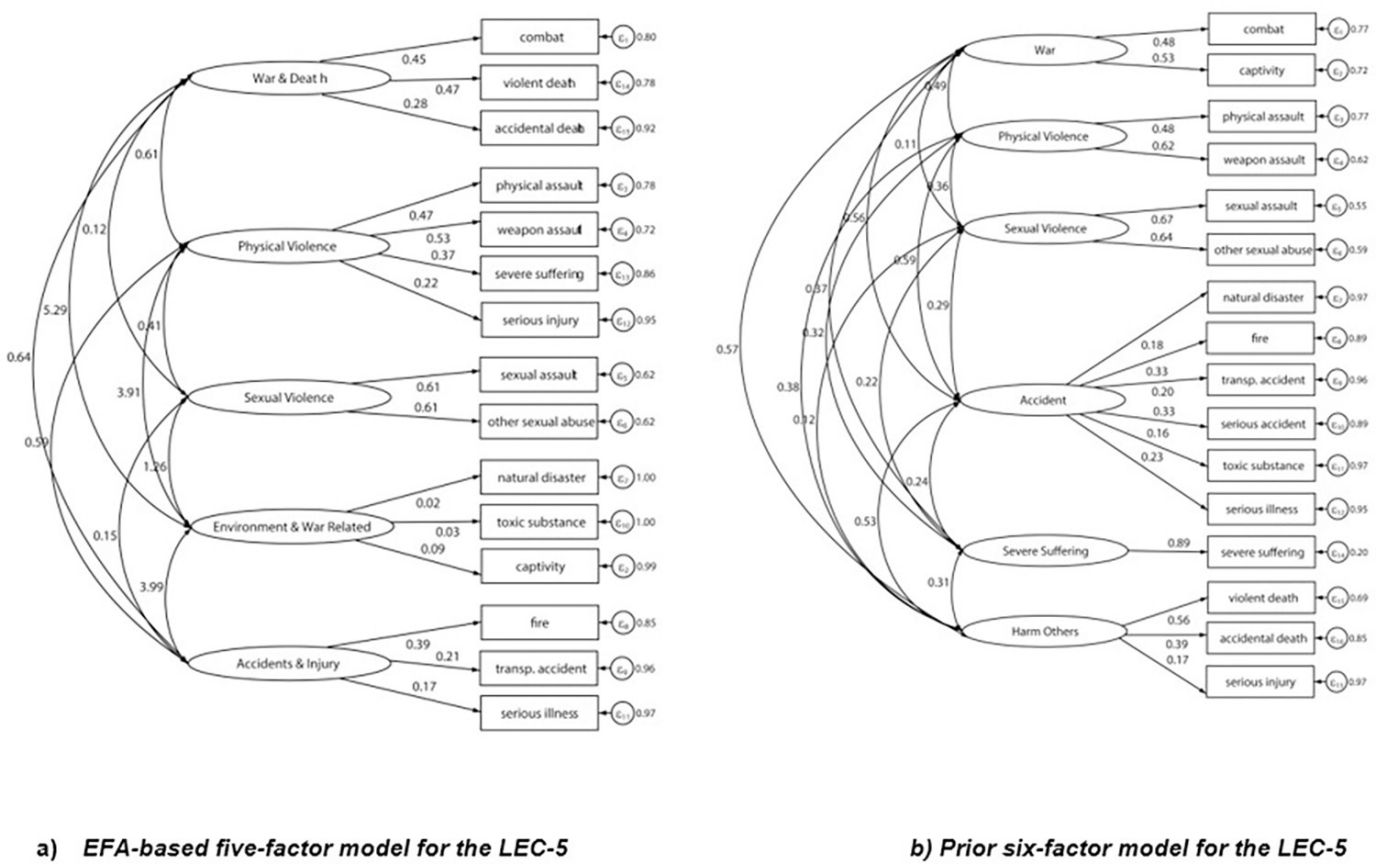
Additional models for Life Events Checklist for DSM-5 (LEC-5) in Uganda. a) EFA-based five-factor model for the LEC-5. b) Prior six-factor mode; for the LEC-5.

Next, we examined the factor structure of the LEC-5 by testing and comparing all three models for fit. The first model was the EFA from this study in the other split-half of our sample. The second model was based on a prior six-factor model and the WHO—World Mental Health Survey findings [2, 26], and the third was a theoretical seven-factor model [33, 34]. CFA models were analyzed with a sample variance-covariance matrix and a maximum likelihood minimization function. Overall, the goodness-of-fit indices showed that the theoretical seven-factor model provided the best fit comparatively (Table 4; Chi-square (85) = 337.08, p < 0.001; SRMR = 0.022, RMSEA = 0.022, TLI = 0.908, CFI = 0.935). In addition, completely standardized parameter estimates from each model solution are presented in Figs 1 and 2. Factor loading estimates showed that items from LEC-5 were strongly related to their specified latent factors in the seven-factor model, except natural disasters (0.18), toxic substance exposure (0.16), and serious illness (0.23), which fell below the cut-off of 0.3 for factor loadings (Fig 1).

**Table 4:** Fit indices for comparison of confirmatory factor analysis models.

	Fit Indices					
	*Χ* ^*2*^	df	*SRMR*	*RMSEA*	*CFI*	*TLI*
**EFA 5 factor**	337.35	80	0.032	0.038	0.855	0.810
**Prior 6 factor**	393.19	90	0.024	0.027	0.922	0.896
**Theory 7 factor**	337.08	85	0.022	0.026	0.935	0.908

All chi-square values are significant at p < .001; SRMR = standardized root-mean-square residual; RMSEA = root mean square error of approximation; CFI = comparative fit index; TLI = Tucker–Lewis index.

## Discussion

The prevalence of experiencing at least one traumatic event among all study participants was 60.5%. We conducted an EFA for a random split-half of the sample, which showed a five-factor solution with three items (combat, severe suffering, and fire/explosion) cross-loading on more than one factor and one item, serious accidents, falling below the 0.3 cut-off for all five factors. A possible explanation for this may be in the way that “serious accidents” are interpreted by the participants which may be different from the construct being assessed. In psychometric analysis, theoretical constructs vary in complexity and some are inherently more intricate and multifaceted than others. Differences in the understanding and interpretation of these constructs may yield lower factor loadings. We then conducted a confirmatory factor analysis to establish an adequate measurement model. These metrics were measured against the six-factor model based on the South Korean EFA of the LEC combined with the WHO—World Mental Health Survey findings [2, 26] and the theoretical seven-factor model used in the South African Stress and Health Survey [33, 34]. The EFA for the LEC-5 in the Ugandan setting showed the poorest model fit for the Ugandan adult population of the three models and indices that did not meet criteria for most cut-offs. These five categories were: sexual violence, war and death, physical violence, environment and war-related events, and accidents and injury. These categories are similar to the six-factor model based on the South Korean EFA of the LEC combined with the WHO—World Mental Health Survey findings [2, 26]. The results of the CFA found that the theoretical seven-factor model was the best fitting model for the present study.

The prevalence of experiencing any traumatic event in our study is lower than in a prior study conducted in northern Uganda, where 80.8% of participants reported experiencing at least one PTE [8]. Mugisha *et al*. used a community sample and the Childhood Trauma Questionnaire (CTQ)–Short Form [36] to assess exposure to traumatic events before the age 18 years old. Their reported prevalence of traumatic events was higher than in our study (even though we include a lifetime timeframe and half of our sample is a clinical population). One possible explanation for the difference in prevalence may be the number of items in each measure with the CTQ which includes 28 items, meaning more opportunities for affirmative answers. Another factor accounting for the prevalence difference may be study’s geographical area, with their study solely taking place in a post-conflict region in northern Uganda [8], and our study taking place across several regions in Uganda. Our study results can also be compared with prior studies conducted in similar settings. For instance, in Ethiopia, researchers examining data from our parent study found a much lower percentage of 48.7% reported exposure to at least one traumatic event [27], whereas in South Africa 92% of participants had one or more traumatic events in their lifetime [27]. Also in South Africa, Atwoli *et al*. found 73.8% using the World Mental Health Composite International Diagnostic Interview (WMH-CIDI; [33, 37]. In Kenya, Kwobah *et al* also using the LEC-5 as part of the NeuroGAP study found that 64.4% of cases reported having experienced at least one PTE in their lifetime [6], which was similar to our findings. Also, in Kenya, Jenkins and colleagues using a structured epidemiological assessment, found 48% of study participants reported traumatic events [38], or lower than in the present study. In high-income countries such as Norway, 86% of women and 85% of men reported experiencing one significant lifetime event [4]. Such variability between and within countries is likely due to differences in the populations studied and measurement instruments used. For example, Jenkins *et al* used open-ended questions with seven examples, which may have elicited fewer endorsements of traumatic events than the LEC-5 used in the Kenyan sample or our sample as part of NeuroGAP-Psychosis. Other possible reasons for the observed heterogeneity in prevalence estimates than differences in assessment scales may be attributed to differences in risk of exposure to PTEs as well as types of PTE (e.g., accidents versus war exposure) [39], definitions of PTE, and time frame references for assessing PTEs [40, 41].

The prevalence of traumatic events in this sample was higher among cases (64.2%) than in controls (55.4%). This finding is similar to other studies across varied settings [42–45]. Also, cases reported more physical and sexual assault than controls, among other traumas. This finding is reflected in studies in Uganda that report a high prevalence of sexual assault and abuse [46, 47], which is consistent with research that has highlighted violent victimization of persons with severe mental illness such as psychosis [48–51].

A theoretical seven-factor model fits our data comparatively well in the present study. This model included the following latent categories: war, physical violence, sexual violence, accidents, network trauma, severe suffering, and witnessing. These groupings of traumatic events from prior research may fit our data well since traumatic events serve less as latent factors and more as composite phenomena [21, 52]. Our findings will probably facilitate the comparability of our results with other studies that utilize similar scales and may help identify and bridge the gap between theoretical constructs and clinical observations specific to the Ugandan setting. Based on the theoretical model, severe injury and severe suffering had the strongest loadings, while items aggregating under the accident latent factor were least strongly associated with it. Sexual assault and other sexual abuse were also strongly related to the sexual violence latent trait. Most Ugandan cultures engage in early marriages, which sets the premise for sexual assault and abuse, especially among women [53]. This may explain the high frequency of sexual assault reported among the participants. Early marriage refers to formal or informal unions in which the girl is less than 18 years [54]. In Uganda, 10% of girls are married before age 15, and 40% will be married before their 18^th^ birthday [55]. Marriage and cohabiting are also important aspects related to sexual violence in Uganda, primarily due to the significance that sexual relationships are given and the roles of spouses in fulfilling sexual obligations [56, 57].

## Strengths and limitations

The strengths of this study include that it is the first study to assess the psychometric properties of the LEC-5 in Uganda in a large sample from different regions and using a standard administration of the LEC-5. However, some limitations should be considered when interpreting the results. First, our sample was comprised of both participants with psychosis and controls, and as such, there may have been an overrepresentation of the types of PTEs among the cases that do not align with the general population, limiting the generalizability of our findings. We do not believe this to be the case, given that the theoretical model fits better than the data driven EFA model, suggesting the PTEs are grouping together in a similar manner as other populations and not simply determined by the makeup of our study sample.

In addition, retrospective reports of PTEs could be biased due to forgetting over time and reality distortion experienced by many patients with psychosis [58, 59]. Moreover, we did not consider cultural variations in perceptions of trauma (e.g., [60] and whether participants perceived their experiences as traumatic. Furthermore, our findings on sexual assault may be underreported as sexual assault and abuse are considered taboo in Ugandan culture, and survivors of abuse who come forward are likely to face social stigma [61, 62].

## Conclusion

The findings from our study support the LEC-5 as a reasonable tool for assessing PTEs in the Ugandan context. This is based on the comparability of our EFA and CFA metrics to the theoretical seven-factor model used in the South African Stress and Health Survey. Therefore, the LEC-5 can be utilized as a screening tool for PTEs in clinical populations. This may aid clinicians in carrying out trauma-related assessments and subsequently providing patient-focused treatment. Further studies should assess the criterion validity and reliability of the LEC-5 in Uganda.
